# Correlates of Hallucinatory Experiences in the General Population: An International Multisite Replication Study

**DOI:** 10.1177/0956797620985832

**Published:** 2021-06-04

**Authors:** Peter Moseley, André Aleman, Paul Allen, Vaughan Bell, Josef Bless, Catherine Bortolon, Matteo Cella, Jane Garrison, Kenneth Hugdahl, Eva Kozáková, Frank Larøi, Jamie Moffatt, Nicolas Say, David Smailes, Mimi Suzuki, Wei Lin Toh, Todd Woodward, Yuliya Zaytseva, Susan Rossell, Charles Fernyhough

**Affiliations:** 1 Department of Psychology, Northumbria University; 2 Department of Clinical and Developmental Neuropsychology, University of Groningen; 3 Department of Biomedical Sciences of Cells & Systems, University Medical Center, University of Groningen; 4 Department of Psychology, University of Roehampton; 5 Department of Psychosis Studies, Institute of Psychiatry, Psychology and Neuroscience, King’s College London; 6 Research Department of Clinical, Educational and Health Psychology, University College London; 7 Department of Biological and Medical Psychology, University of Bergen; 8 Laboratoire Inter-Universitaire de Psychologie, Université Grenoble Alpes; 9 Department of Psychology, Institute of Psychiatry, Psychology and Neuroscience, King’s College London; 10 South London & Maudsley NHS Foundation Trust, Maudsley Hospital, London, England; 11 Department of Psychology, University of Cambridge; 12 Division of Psychiatry, Haukeland University Hospital, Bergen, Norway; 13 Department of Applied Neurosciences and Brain Imaging, National Institute of Mental Health, Klecany, Czech Republic; 14 Department of Psychology, Faculty of Arts, Charles University; 15 Psychology and Neuroscience of Cognition Research Unit, University of Liege; 16 NORMENT–Norwegian Center of Excellence for Mental Disorders Research, University of Oslo; 17 School of Psychology, University of Sussex; 18 Division of Psychiatry, University College London; 19 Cognitive Neuropsychiatry Lab, Centre for Mental Health, Swinburne University of Technology; 20 Department of Psychiatry, The University of British Columbia; 21 BC Mental Health and Substance Use Services, Provincial Health Services Authority, Vancouver, British Columbia, Canada; 22 Department of Psychiatry and Medical Psychology, Third Faculty of Medicine, Charles University; 23 Department of Psychiatry, St Vincent’s Hospital, Melbourne, Australia; 24 Department of Psychology, Durham University

**Keywords:** cognitive processes, auditory perception, language, memory, hallucinations, open materials, preregistered

## Abstract

Hallucinatory experiences can occur in both clinical and nonclinical groups. However, in previous studies of the general population, investigations of the cognitive mechanisms underlying hallucinatory experiences have yielded inconsistent results. We ran a large-scale preregistered multisite study, in which general-population participants (*N* = 1,394 across 11 data-collection sites and online) completed assessments of hallucinatory experiences, a measure of adverse childhood experiences, and four tasks: source memory, dichotic listening, backward digit span, and auditory signal detection. We found that hallucinatory experiences were associated with a higher false-alarm rate on the signal detection task and a greater number of reported adverse childhood experiences but not with any of the other cognitive measures employed. These findings are an important step in improving reproducibility in hallucinations research and suggest that the replicability of some findings regarding cognition in clinical samples needs to be investigated.

Hallucinations are often associated with a diagnosis of schizophrenia (Bauer et al., 2011) or other psychiatric disorders (Toh et al., 2016), but they can also occur in people who have not been diagnosed at all (Powers et al., 2017; Sommer et al., 2010). Consistent with a dimensional or continuum view of psychosis (van Os et al., 2000), findings have shown that susceptibility to hallucinatory experiences varies across the population (Siddi et al., 2019). This has led researchers to propose the existence of a psychosis phenotype, or a continuous-hallucination phenotype (Aleman & Larøi, 2008). Such hallucinatory experiences are assumed to share at least some phenomenological, etiological, and cognitive components with hallucinations in psychiatric disorders (but see David, 2010). Investigating associated cognitive mechanisms in the general population is crucial because it avoids confounding variables (e.g., use of antipsychotic medication) while allowing the development of mechanistic models that can account for both unusual nonclinical experiences and distressing experiences in psychosis. Such models are also informative regarding the nature of agency and perception. However, in studies of hallucinatory experiences, there has been little focus on reproducibility and replication, and contradictory findings are common in the field.

For example, some studies have found that hallucinations in psychosis are associated with a bias in source monitoring—when a self-generated cognition is misattributed to an external source (e.g., Woodward et al., 2007; Gaweda et al., 2013). A number of studies have shown a similar link between source monitoring and hallucinatory experiences in the general population (e.g., Larøi et al., 2004), whereas other studies have shown no such link (Alderson-Day et al., 2019). Other studies have used auditory signal detection tasks to assess the role of top-down processing in hallucinatory experiences, requiring psychosis patients with hallucinations to detect short speech clips embedded in bursts of noise (Brookwell et al., 2013). A number of studies have reported an increase in false-alarm responses in participants who report more hallucinatory experiences (Barkus et al., 2011; Varese et al., 2012). Nevertheless, there are inconsistent results regarding whether this is associated with a lower response threshold (the criterion for accepting the presence of a stimulus) and/or lower task accuracy, as well as suggestions of publication bias in this area (Brookwell et al., 2013).

Research into language lateralization and attentional control using a consonant-vowel dichotic-listening task has also provided evidence for links with hallucinations. In this task, participants must discriminate conflicting speech stimuli presented simultaneously to both ears; participants typically exhibit a right-ear advantage (Bless et al., 2015). Meta-analytic evidence shows that psychosis patients with hallucinations do not show this response pattern (Ocklenburg et al., 2013), although again, studies are inconsistent regarding whether this pattern is linked to hallucinatory experiences in the general population (Aase et al., 2018; Conn & Posey, 2000). Similarly, reduced verbal working memory is frequently reported in schizophrenia and may be further impaired in hallucinating patients (Gisselgård et al., 2014). Some studies have noted poorer verbal working memory in individuals in the general population who report more frequent psychotic-like experiences (e.g., Rossi et al., 2016), although other studies found no such association with schizotypy (Barkus et al., 2011). Indeed, one potential reason for inconsistency may relate to variation in the scales used, including broader assessments of psychotic-like experiences or hallucination proneness, or focuses on specific modalities of hallucination. Regarding environmental factors, the literature is more consistent in linking childhood trauma with hallucinations in both psychosis (Bailey et al., 2018) and hallucinatory experiences in the general population (Lataster et al., 2006).

**Statement of Relevance**

Hallucinations are often reported by people with psychiatric diagnoses such as schizophrenia, but they also sometimes occur in people in the general population who have never been diagnosed with a psychiatric disorder. A number of previous studies have found that the propensity to report hallucinations is associated with aspects of cognition such as biases in speech and language detection as well as impairments in some types of memory. However, many of these studies have not been replicated, and some were conducted with only small numbers of participants, meaning that previous findings might not be reliable. Our study assessed hallucinatory experiences in a large number of participants across a number of data-collection sites around the world. We found that only biases in speech detection were linked to hallucinations, calling into question other findings regarding links among language, memory, and hallucinations. We recommend that practices such as study replication and data sharing be more commonly used in this research area.

In addition to inconsistent results, there are few standardized procedures, and sample sizes have been small (a mean of 23 per group in one meta-analysis; Brookwell et al., 2013), limiting power and potentially overestimating effect sizes (Button et al., 2013). Coupled with the lack of open-science practices (Tackett et al., 2019), including a lack of preregistration, replication, and openly available data and materials, there should be serious concerns regarding the reproducibility of findings in this research area. We sought to address this using the many-labs model developed by Klein et al. (2014). We collected behavioral task data and assessed participants for hallucinatory experiences across 11 data-collection sites, as well as recruiting online. The aim was to recruit participants across the continuum of hallucinatory experiences and collect a large enough sample to provide the ability to detect small effect sizes. Because of methodological variability in the previous literature, we created a single centralized test battery used by all participating research groups. Participants completed assessments of hallucinatory experiences; source-memory, dichotic-listening, verbal working memory, and auditory signal detection tasks; and an assessment of adverse childhood events. Given the recent focus on the prevalence and quality of online data collection (de Boer et al., 2019; Peer et al., 2017), we also sought to investigate the quality of data gained through online collection.

Our hypotheses, presented in Table 1, focused on key empirical results that have been used to support central conclusions about the cognitive mechanisms of hallucinatory experiences.

**Table 1.** Summary of Hypothesis for Each Measure

Hypothesis	Construct assessed	Variable of interest	Key reference
Hypothesis 1 (source memory): The number of imagined words incorrectly recalled as heard should be positively associated with hallucinatory experiences.	Verbal source monitoring	Number of externally misattributed words	Larøi et al. (2004); Alderson-Day et al. (2019)
Hypothesis 2 (dichotic listening): The number of correct right-ear responses in the nonforced condition and the number of correct left-ear responses in the forced-left condition should be negatively associated with hallucinatory experiences.	Language lateralization, attentional control	Number of correctly reported right- or left-ear syllables	Conn & Posey (2000); Aase et al. (2018)
Hypothesis 3 (backward digit span): Mean digit span should be negatively associated with hallucinatory experiences.	Verbal working memory	Mean digit span	Barkus et al. (2011); Rossi et al. (2016)
Hypothesis 4 (auditory signal detection): False alarms should be positively associated with hallucinatory experiences.	Top-down processing on speech	Number of false alarms	Barkus et al. (2011); Varese et al. (2012)
Hypothesis 5 (adverse childhood experiences): This score should be positively associated with hallucinatory experiences.	Adverse childhood experiences	Number of adverse childhood experiences reported	Janssen et al. (2004); Lataster et al. (2006)
Hypothesis 6 (for data collected online): Effect size should differ for participants who failed all attention checks compared with participants who passed at least two thirds of the attention checks.	Quality of online data and success of attention checks	Quality of online data and success of attention checks	Peer et al. (2017)

## Method

### Preregistration

The study protocol, hypotheses, variables of interest, exclusion criteria, and sample size were preregistered on AsPredicted.org (https://osf.io/cyu6j) on February 27, 2018, before data collection commenced. One deviation from the preregistration and additional nonpreregistered analyses are detailed in the Results section.

### Participants

Participants were recruited via two methods: (a) lab data collection (i.e., participants attended a data-collection site and took part in the study under laboratory conditions) and (b) online data collection (i.e., participants were recruited online and completed the tasks on their own computer). Previous meta-analyses of comparable general-population studies have shown large effect sizes in this research area. For example, Brookwell et al. (2013) reported a *g* of 0.8 (95% confidence interval, or CI = [0.54, 1.06]). Converting the lower CI in this estimate to *r* would give an effect size of .26. Our main aim was to collect as large a sample as possible at as many data-collection sites as possible, so decisions regarding sample size were not based purely on power analyses; we preregistered a minimum sample size of 420 for lab-based data collection (based on the anticipated number of data-collection sites) and 800 for online data collection (based on available funding). Given only our anticipated lab-based sample, 420 participants would allow a power of .80 to detect a small effect size (*r*) of .12—although our aim was to collect substantially more than this number. The final sample size was 1,513 (647 in the lab, 866 online) before exclusions. The sample size after exclusion criteria were applied was 1,394 (see the Results section below as well as the Supplemental Material available online). Demographic information can be found in Table 2 and in Results.

**Table 2.** Demographic Characteristics of the Sample (*N* = 1,394) and Association Between Each Variable and the Cardiff Anomalous Perceptions Scale (CAPS) Score

Variable	Mean or percentage of sample	Association with CAPS score	95% CI
Age (years)	*M* = 29.4 (*SD* = 10.9)	*r*_S_ = −.17	[−.11, −.22]
Gender (female)	55.7%	*d* = 0.05	[−0.06, 0.16]
Handedness (left)	10.8%	*d* = −0.01	[−0.18, 0.16]
Diagnosis	16.0%	*d* = −0.55	[−0.70, −0.41]
Relative diagnosis	19.5%	*d* = −0.28	[−0.41, −0.15]
Cigarette usage	16.2%	*r*_S_ = .052	[.003, .11]
Alcohol intake	56.0%	η_*p*_^2^ = .005	[.00, .011]
Cannabis usage	8.6%	η_*p*_^2^ = .024	[.011, .037]
Parental income	14.5%	η_*p*_^2^ = .006	[.00, .012]

Note: The percentage for diagnosis includes participants who reported any form of psychiatric or neurological diagnosis. Relative diagnosis includes participants who reported having first-degree relatives with any form of psychiatric or neurological diagnosis. Cigarette usage includes participants who reported smoking at least one cigarette per day. Alcohol intake includes participants who reported drinking alcohol at least twice per month. Cannabis usage includes participants who reported using cannabis at least twice per month. Parental income includes participants who reported that their parents had less than enough money to meet the family’s needs during childhood. Note that confidence intervals (CIs) for η_*p*_^2^ cannot cross 0 (because η_*p*_^2^ cannot be a negative value).

### Lab data-collection sites

The study was advertised as part of a working group of the International Consortium for Hallucinations Research. Participating sites were required to recruit a minimum of 40 participants into the study to be eligible for inclusion in the final data set. Twelve sites were involved in data collection, situated in the United Kingdom (six sites), France, The Netherlands, Czech Republic, Norway, Canada, and Australia (one site per country). All sites obtained ethical clearance from their relevant institutional review board in accordance with the Declaration of Helsinki. Participants were required to be between the ages of 18 and 75 years, fluently speak the native language of the respective country, and report no diagnosed hearing impairments. Participants were given a small reward for participation at the discretion of each participating site (e.g., a gift voucher, course credits, a small payment, a prize-draw entry).

### Online data collection

In addition to data collection in labs at participating sites, the study was also advertised on the website Prolific Academic (prolific.ac), a recruitment website through which researchers can advertise online behavioral studies and reward participants with small payments for task and questionnaire completion. Eligibility criteria and exclusion criteria were the same as for the lab-based data. Participants were rewarded with a payment of £4.20 for participation.

### Task platform

All tasks and questionnaires were programmed in JavaScript using the *jsPsych* toolbox (Version 6.1; de Leeuw, 2015) and run from an Internet browser (code accessible at https://osf.io/eqy76/). For the purpose of this study, all measures were translated and back-translated from English into French, Czech, and Norwegian for use at data-collection sites in countries where these were the primary language, and verbal stimuli suited to each language were used for the source-memory and dichotic-listening tasks.

For online data collection, participants were required to complete a task designed to ensure that they were wearing headphones (developed by Woods et al., 2011) before gaining access to the main task platform (see Section S1 in the Supplemental Material). Additional attention checks are described below.

### Questionnaires

#### Cardiff Anomalous Perceptions Scale (CAPS)

The CAPS (Bell et al., 2006) was employed as the primary assessment of hallucinatory experiences. It consists of 32 items (e.g., “Do you ever hear noises or sounds when there is nothing about to explain them?”) with “yes” and “no” as response options. The primary outcome variable, as specified in the preregistration, consisted of the total number of items on which the participant responded “yes” (scored as 1, so scores varied from 0 to 32, with higher values indicating higher levels of hallucinatory experiences). Further subscales on distress, intrusiveness, and frequency were included but not used in any preregistered analysis.

#### Launay-Slade Hallucination Scale-Extended (LSHS-E)

The LSHS-E (Larøi et al., 2004) was employed as a secondary assessment of hallucinatory experiences because of its frequent use in studies examining hallucinatory experiences in the general population. It consists of 16 items (e.g., “I have been troubled by hearing voices in my head”), and participants are asked to respond on a 5-point Likert-type scale (0 = *certainly does not apply to me*, 4 = *certainly applies to me*); the overall score is calculated as the sum of the score for each item (0–64). Compared with the CAPS, the LSHS-E assesses a range of more commonly reported experiences, including intrusive thoughts and vivid daydreams, as well as multisensory and auditory-visual hallucinatory experiences.

#### Adverse Childhood Experiences (ACE) scale

The ACE scale (Felitti et al., 1998) was used as an assessment of childhood trauma. It consists of 17 items (e.g., “Did a parent or other adult in the household often or very often swear at, insult, or put you down?”), and participants respond “yes” or “no” for each item. The total score was calculated as the sum of “yes” responses (0–17).

#### Additional measures

Two further scales were included not to test any specific hypotheses but simply to characterize the sample and for potential exploratory analysis: the Schizotypal Personality Questionnaire (Davidson et al., 2016) and the Depression Anxiety and Stress Scale (Lovibond, 1998). No analysis was conducted using these scales in this article. Participants also provided basic demographic information and answered questions regarding their alcohol, nicotine, and cannabis intake.

#### Attention checks

Three questions taken from the study by Peer et al. (2017) were included in the questionnaires on the task platform. These questions were designed to be easily answerable and thus acted as attention checks. Participants were excluded from all data analysis if they incorrectly answered more than one attention-check question (see Section S2 in the Supplemental Material).

### Source-memory task

The source-memory task required participants to recall whether words had been presented as spoken stimuli through headphones (*hear* trials) or whether they had been instructed to imagine hearing the words (*imagine* trials).

In the first stage of the task, participants were presented with a series of words in the center of the screen (duration = 3 s), each preceded by the word “HEAR” or “IMAGINE” (duration = 1 s). For trials on which participants heard the stimuli, a word from the hear condition was presented in the center of the screen, and an audio clip of that word spoken by a man, in a neutral tone, was presented concurrently. For trials on which participants were instructed to imagine the word, a word from the imagine condition was presented on the screen, but no speech clip was played. The second stage of the task began immediately after the first was completed. Participants were shown all 48 words from Stage 1 in random order, as well as 24 new words. For each word, they were instructed to decide whether they had heard the word, whether they had imagined the word, or whether the word was new. The primary variable of interest in this task was the number of responses on which the participant mistakenly decided that they had heard a word from the imagine list (imagine-to-hear errors).

The task was based on previously used versions (e.g., Moseley et al., 2018), although it differed from others in a number of ways to ensure consistency across data-collection sites and online. For example, participants listened to recordings of a voice rather than to an experimenter reading the word aloud. Some previous tasks have also required participants to generate their own verbal stimuli (Larøi et al., 2004) or complete word pairs (Alderson-Day et al., 2019), whereas the task used here presented single words via recording.

### Consonant-vowel dichotic listening

The dichotic-listening task is designed to assess language lateralization with two additional forced-attention conditions to assess cognitive or attentional control. The task used stimuli that were identical to those in previous studies (e.g., Aase et al., 2018; Hugdahl et al., 2013). The task involves the simultaneous presentation of two audio clips of spoken consonant-vowel syllables, with a different syllable presented to each ear. The presented syllables are “ba,” “da,” “ka,” “ta,” “pa,” and “ga”; each clip lasted approximately 350 ms. In the nonforced-attention condition, participants were required to select the syllable that they could hear most clearly. In the forced-right and forced-left conditions, participants were instructed to select the syllable that they believe had been presented to the right or left ear, respectively. Participants provided a response with a mouse click.

There were 36 trials in each condition, presented in a random order, including six homonym trials (with the same syllable presented to each ear). The homonym trials were excluded from data analysis and used only as a data-quality check. Resulting variables were the total number of correctly identified syllables presented to the right ear (REC; for the nonforced and forced-right conditions) or correctly identified syllables presented to the left ear (LEC; forced-left condition only). The laterality index, [(REC − LEC)/(REC + LEC)] × 100, was calculated for further analysis.

### Backward digit span

The digit-span task assessed verbal working memory performance; each trial required participants to view a series of digits and then recall these digits in reverse order. Previous studies of hallucinatory experiences (e.g., Barkus et al., 2011) have required participants to respond by speaking their answer aloud; here, we used a computerized version of the task that required a response via a mouse click and adaptively increased or decreased the length of the digit string on the basis of performance, as recommended by Woods et al. (2011). Digits (1–9) were presented in the center of the screen, randomly sampled without replacement (until trial length of 10 digits, when the numerals were resampled). Each digit was presented on screen for 1 s. Trial length started at two digits and was varied according to the rules set out by Woods et al. (2011); that is, a correctly recalled digit string led to an increase in digit length by one, whereas two consecutive incorrectly recalled digit strings decreased the digit length by one. Participants used an on-screen keypad to click on the digits they wanted to input. All participants completed 14 trials. Performance was assessed using the mean-span method described by Woods et al. (2011), which estimates the digit length at which the participant performs with 50% accuracy.

### Auditory signal detection (lab data collection only)

The auditory signal detection task required participants to respond as to whether they believed that a speech clip had been embedded in noise. The task was identical to that in previous studies (e.g., Barkus et al., 2011). The signal-to-noise ratio (SNR; i.e., the ratio of the volume of the voice clip to the noise) was determined individually at each site using a short calibration task with participants who did not participate in the main study (*n* = 10 per site). This task was administered only with participants in the lab because calibration would not have been possible with online participants.

In the main task, the participant was presented with seventy-two 3.5-s bursts of pink noise. In the middle of 36 of these trials, a 1.5-s speech clip was presented at one of four SNRs (speech present); in the other 36 trials, no speech clip was presented (speech absent). The speech clips, which were the same as those used in previous studies employing this task (Barkus et al., 2011), consisted of a male voice reading text (taken from an instruction manual) in an emotionally neutral tone. After each burst of noise, participants were presented with the text, “Did you hear speech?” and responded by clicking a mouse button for “yes” or “no.” For each trial, they were also then prompted to enter a confidence rating, data from which will be analyzed and reported in a future article. The primary outcome variable was false-alarm rate (the percentage of voice-absent trials on which the participant incorrectly responded that a speech clip was present). Secondary outcome variables were hit rate, task sensitivity ( d′ , calculated as the standardized false-alarm rate subtracted from the standardized hit rate), and criterion β ( $\beta =e\left\{\frac{Z{\left(FA\right)}^{2}-Z{\left(H\right)}^{2}}{2}\right\}$ ; also known as response bias).

### Matrix reasoning

This task was included to provide a brief assessment of nonverbal reasoning ability. Ten items were taken from the International Cognitive Ability Resource (previously tested in more than 97,000 participants; Condon & Revelle, 2014). Participants completed a 3 × 3 grid of shapes, choosing from six options, within 60 s. The raw number of correct responses was used as a measure of nonverbal reasoning ability.

### Procedure

For participants in a lab environment, testing took place in a quiet room at a laptop or desktop computer using over-ear headphones for tasks involving auditory stimuli. The study took approximately 50 to 60 min to complete. The task platform presented the dichotic-listening, source-memory, matrix-reasoning, digit-span, and auditory signal detection tasks, followed by questionnaire measures. The task platform used in online data collection was identical, with the exception being the inclusion of a headphone-check task (see Section S1 in the Supplemental Material) and exclusion of the auditory signal detection task, which relied on laboratory-controlled conditions.

### Data analysis

Exclusions based on preregistered criteria (e.g., poor task performance) are outlined in Section S3 in the Supplemental Material. First, we examined associations between demographics and CAPS score and measures of alcohol, cannabis, cigarette usage, and nonverbal reasoning. These analyses were not preregistered and included for descriptive purposes.

To assess associations between task performance and CAPS score, as detailed in Hypotheses 1 through 5, we calculated simple correlations (Spearman’s *r* [*r*_S_] for nonnormally distributed variables) with associated 95% CIs. For the preregistered analyses, when CIs included 0 (indicating a potential null effect), equivalence testing was conducted with upper and lower bounds of *r*_S_ (0.1 and −0.1). Equivalence testing was done using the *TOSTER* package (Version 0.3.4; Lakens, 2017) in the R programming environment (Version 4.0.2; R Core Team, 2020). These bounds were chosen as representing small effect sizes; effects significantly smaller than this are likely to be of negligible relevance. When a significant *p* value is reported for an equivalence test, this can be taken to indicate that the effect is indistinguishable from 0 (providing evidence for the null hypothesis).

As well as assessing simple correlations between task measures and CAPS score, we also constructed linear mixed models, with data-collection site as a random effect, task measures as fixed effects, and CAPS score as the dependent variable, to investigate which cognitive-task variables would contribute to the highest quality model. To assess model quality, we used the Akaike information criterion (AIC), a measure that takes into account both predictive ability and number of parameters in a model (with fewer seen as better). All data analysis was conducted in R (code is available at https://osf.io/eqy76/).

## Results

### Sample

**Fig. 1.**
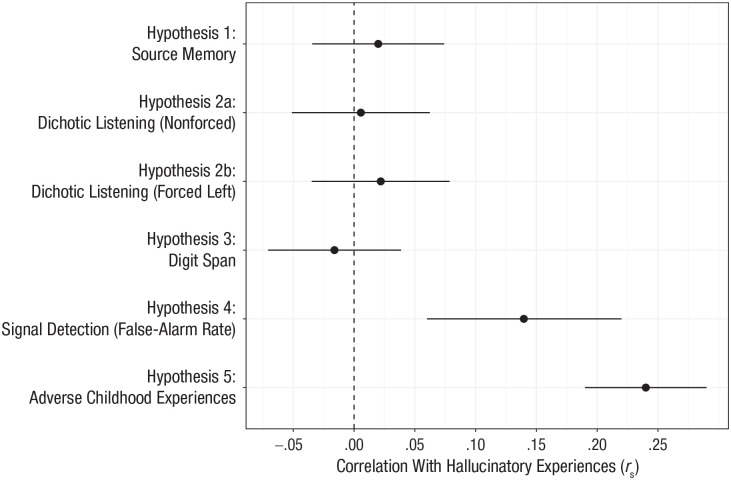
Correlation (Spearman’s *r*) between hallucinatory experiences and the primary outcome variable for each task. Hallucinatory experiences were measured using the Cardiff Anomalous Perceptions Scale. Error bars represent 95% confidence intervals.

In total, 1,513 participants were recruited into the study. One UK-based data-collection site did not meet the minimum sample-size requirement and was therefore not included in any analysis. Of the final sample, 647 participants (42.8%) took part in a laboratory environment, whereas 866 (57.2%) took part in the online version of the study. After we applied the preregistered exclusion criteria, the final sample consisted of 1,394 participants (594 in the lab, 800 online) native to 46 countries. Further demographic information can be seen in Table 2. Correlations for Hypotheses 1 through 5 are presented in Table 3. Figure 1 also shows the finding for each hypothesis.

**Table 3.** Correlation Matrix for Cardiff Anomalous Perceptions Scale (CAPS) Score and Primary Outcome Variables for Each Measure

Variable	1	2	3	4	5	6
1. CAPS score	—					
2. Source memory: imagine-to-hear errors	.019 [−.03, .07]	—				
3. Dichotic listening: nonforced condition	.006 [−.05, .06]	−.038 [−.09, .02]	—			
4. Dichotic listening: forced-left condition	.022 [−.03, .08]	.033 [−.09, .02]	.**126** [.07, .18]	—		
5. Digit span: mean span	−.016 [−.07, .04]	**–.065** [−.12, −.01]	.050 [−.01, .11]	.**071** [.02, .13]	—	
6. Signal detection task: false-alarm rate	.**140** [.06, .22]	.019 [−.06, .10]	.061 [−.02, .14]	.011 [−.07, .09]	.056 [−.03, .14]	—
7. Adverse childhood experiences: number endorsed	.**241** [.19, .29]	−.006 [−.06, .05]	.006 [−.06, .05]	.008 [−.05, .06]	−.050 [−.10, .003]	.011 [−.07, .09]

Note: Correlations are presented as Spearman’s *r* because of nonnormality of variables. Values in brackets are 95% confidence intervals (CIs). Boldface indicates 95% CIs that do not include 0. In the source-memory task, imagine-to-hear errors indexed the number of imagined words misremembered as heard. In the dichotic-listening task, scores in the nonforced and forced-left conditions were the number of correctly identified syllables presented to the right ear and left ear, respectively. In the backward digit-span task, mean span was the measure of verbal working memory. The false-alarm rate in the signal detection task was indexed by the proportion of voice-absent trials on which participants responded “yes.”

### Assessment of hallucinatory experiences

Across the whole sample, participants endorsed a mean of 4.68 items on the CAPS (95% CI = [4.42, 4.93], *Mdn* = 3, range = 0–32). Internal reliability of the CAPS was good ( α = .87). The CAPS score was strongly positively skewed (skewness = 1.58, *SE* = 0.07) and leptokurtic (kurtosis = 3.21, *SE* = 0.13); most participants reported few hallucinatory experiences, and a smaller number of participants reported many hallucinatory experiences. That said, 50 participants scored at or above the mean score of psychosis patients (e.g., Bell et al., 2006, 2011), suggesting that the sample covered a sufficient range of the hallucination continuum. Consistent with previous findings (Bell et al., 2006, 2011), a nonpreregistered analysis showed that the CAPS score was associated with age, having a psychiatric diagnosis, having a first-degree relative with a psychiatric diagnosis, and cannabis usage (see Table 2). There was no association between the CAPS score and nonverbal reasoning, as assessed by matrix reasoning (*r*_S_ = .02, 95% CI = [−.03, .08], *p* = .399). The LSHS-E was used as a secondary measure of hallucinatory experiences, and participants’ mean score was 20.33 (95% CI = [19.71, 20.94], *Mdn* = 20, range = 0–60).

### Hypothesis 1: hallucinations and source memory

We included 1,375 participants’ data for the source-memory task. Overall accuracy was well above chance (*M* = 64.97%, 95% CI = [64.33, 65.60]). In terms of source judgments, participants were more likely to misattribute a heard item as imagined (hear-to-imagine error; *M* = 6.26, 95% CI = [6.08, 6.45]) than to misattribute an imagined item as heard (imagine-to-hear error; *M* = 4.04, 95% CI = [3.88, 4.19]), *t*(1381) = 18.29, *p* < .001, *d* = 0.49.

The number of imagine-to-hear errors (i.e., external misattributions) was used as the primary variable to assess source-monitoring performance (Hypothesis 1). There was no correlation between number of imagine-to-hear errors and CAPS score, *r*_S_(1376) = .02, 95% CI = [−.03, .07], *p* = .461. Equivalence testing indicated that the effect was statistically indistinguishable from 0, given equivalence bounds of −0.1 and 0.1 (*p* = .001). Similarly, further analysis indicated that there was no association between imagine-to-hear errors and score on the LSHS-E, *r*_S_(1377) = −.005, 95% CI = [−.06, .05], *p* = .839.

Further, in an exploratory (nonpreregistered) analysis, we calculated overall *reality-monitoring accuracy*, that is, the proportion of correctly recalled “old” words for which the source was also correctly recalled—as in the study by Garrison et al. (2017). The calculation was as follows: (hear-hear + imagine-imagine)/(hear-hear + imagine-imagine + hear-imagine + imagine-hear) × 100, where hear-hear refers to heard items correctly labeled as heard, hear-imagine refers to heard items incorrectly labeled as imagined, and so on. There was no association between reality monitoring accuracy and CAPS score (*r*_S_ = .04, *p* = .11).

### Hypothesis 2: hallucinations and dichotic listening

We included 1,262 participants’ data for the dichotic-listening task. Across the whole sample, a right-ear advantage was observed; participants also successfully oriented their attention in the forced-left and forced-right conditions, as in previous research (e.g., Bless et al., 2015; see Section S5 in the Supplemental Material).

There was no correlation between CAPS score and performance in the nonforced condition of the dichotic-listening task, as assessed by the number of right-ear responses (Hypothesis 2), *r*_S_(1263) = .006, 95% CI = [−0.05, 0.06], *p* = .842, and equivalence testing indicated that the effect was statistically indistinguishable from 0 (*p* < .001). Similarly, there was no association between CAPS score and the number of correct left-ear responses, *r*_S_(1263) = .022, 95% CI = [−.03, .08], *p* = .435, in the forced-left condition, which was also indistinguishable from 0 (*p* = .003).

In a secondary analysis, total LSHS-E score also showed no association with dichotic-listening performance for all conditions (all *r*_S_ < .019, *p*s > .493).

### Hypothesis 3: hallucinations and verbal working memory

Overall mean span (*M* = 6.39, 95% CI = [6.31, 6.47]) was approximately equal to that reported by Woods et al. (2011). There was no association between mean digit span and CAPS score (Hypothesis 3), *r*_S_(1358) = −.02, 95% CI = [−.07, .04], *p* = .552, and equivalence testing indicated an effect indistinguishable from 0 (*p* < .001), although secondary analysis showed a very weak association between mean digit span and LSHS-E score, *r*_S_(1357) = −.06, 95% CI = [−.11, −.0004], *p* = .042.

### Hypothesis 4: hallucinations and auditory signal detection

Auditory signal detection data were collected only from participants who took part in the lab-based version of the study (*n* = 594). The mean hit rate was comparable with that in previous studies using this task (*M* = 74.39%, 95% CI = [73.18, 75.59]), as was the false-alarm rate (*M* = 23.29%, 95% CI = [21.44, 25.15]).

There was a positive association between CAPS score and false-alarm rate (Hypothesis 5), *r*_S_(581) = .14, 95% CI = [.06, .22], *p* < .001. Additional analysis also showed a positive association between CAPS score and hit rate, *r*_S_(581) = .18, 95% CI = [.10, .26], *p* < .001, and a negative association between CAPS score and β, *r*_S_(581) = −.17, 95% CI = [−.25, −.09], *p* < .001, indicating that increased CAPS score was associated with a reduced threshold for accepting the presence of a stimulus. There was no such association between CAPS score and *d*′, *r*_S_(581) = −.05, 95% CI = [−.13, .03], *p* = .238, although equivalence testing indicated that the correlation was not statistically equivalent to 0 (*p* = .110).

Using LSHS-E as a secondary outcome, we observed similar associations with false-alarm rate, *r*_S_(581) = .12, 95% CI = [.03, .19], *p* = .005, and β, *r*_S_(581) = −.12, 95% CI = [−.20, −.04], *p* = .005. Unlike with the primary outcome measure, there was also a small association between LSHS-E score and *d*′, *r*_S_(581) = −.10, 95% CI = [−.18, −.02], *p* = .018.

### Hypothesis 5: hallucinations and adverse childhood events

The mean number of adverse childhood experiences reported was 1.75, although this was heavily positively skewed, with a median of 1; 53.9% of participants reported one or more adverse childhood experiences.

There was a positive correlation between CAPS total and ACE score, *r*_S_(1366) = .24, 95% CI = [.19, .29], *p* < .001. A similar effect size was found when LSHS-E was used as a secondary outcome measure to assess hallucinations, *r*_S_(1366) = .24, 95% CI = [.19, .29], *p* < .001.

### Hypothesis 6: attention checks and data quality

Because of a low number of participants failing all attention checks (*n* = 15), we diverged from our preregistered analysis plan and compared participants who failed two or more checks—and were hence excluded from the full analysis (the *failed*-*checks* group, *n* = 66)—with those who failed one check or fewer (the *included* group). The failed-checks group scored lower on all primary outcome variables, although CIs included 0 in all cases other than mean digit span (*U* = 37,728, 95% CI for difference in means between groups = [0.165, 1.004], *p* = .004, *d* = 0.34). Correlation coefficients were also computed between task variables and CAPS score for the failed-checks group only and compared with the coefficients gained in the main analysis. There were no differences between the two groups in correlation coefficients (all 95% CIs overlapped). For a comparison of data collected in the lab compared with online, see Section S6 in the Supplemental Material.

### Constructing a model to predict hallucinatory experiences from cognitive task performance

We constructed three linear mixed models (using the *lme4* package in R; Bates et al., 2015), each with CAPS score as the dependent variable and data-collection site as a random effect (intercept). This analysis was conducted on data collected only in the lab so signal detection data could be included. Predictor variables were centered and standardized. Assumptions regarding multicollinearity, homoscedasticity, homogeneity of variance, and normality of random effects were met. However, inspection of quantile-quantile (QQ) plots suggested nonnormality of residuals; therefore, CAPS score was log transformed, and the models were recomputed. For these models, QQ plots suggested normality of residuals; therefore, the models with a log-transformed CAPS score were used.

The first model (baseline) included basic demographic information (age, gender, and parental income) as fixed effects, which significantly improved on a model with only the random effect entered (*p* < .001). The second model (signal detection) added the false-alarm rate in the signal detection task as a fixed effect and significantly improved on the baseline model (*p* = .028). The third model added the remaining task variables (right-ear syllables in the nonforced condition of the dichotic-listening task, mean span in the digit-span task, imagine-to-hear errors in the source-memory task) and did not improve on the signal detection model (*p* = .965). The AIC was also used to assess model quality. The signal detection model provided the lowest AIC (change in AIC = 470.5). See Table 4 for the full coefficients for the model in which all variables were included and Section S8 in the Supplemental Material for the full breakdown of model comparisons.

**Table 4.** Coefficients for the Linear Mixed Model Containing Variables From All Task Measures

Fixed effect	*b*	*SE*	β	β *SE*	*t*	*p*
Intercept	0.68	0.071			*t*(280.0) = 9.42	< .001
Age	−0.07	0.018	−0.17	0.045	*t*(493.7) = −3.80	< .001
Gender	0.01	0.017	0.03	0.042	*t*(529.3) = 0.68	.499
Parental income	−0.03	0.020	−0.07	0.043	*t*(534.9) = −1.56	.120
Signal detection	0.04	0.016	0.09	0.042	*t*(534.2) = 2.20	.028
Dichotic listening	0.003	0.017	0.01	0.043	*t*(506.6) = 0.20	.842
Source memory	0.01	0.017	0.02	0.042	*t*(528.5) = 0.41	.679
Digit span	−0.004	0.017	−0.01	0.042	*t*(534.1) = −0.23	.815

Note: The dependent variable in this model was log-transformed Cardiff Anomalous Perceptions Scale (CAPS) score, and the random effect was data-collection site (variance = 0.005, *SD* = 0.07); *p* values for fixed effects were calculated using Satterthwaite’s approximations. The linear mixed model was calculated using the *lme4* package in R (Bates et al, 2015). The model equation was specified as follows: CAPS total score ~ age + gender + parental income + signal detection task false alarms + dichotic-listening task right-ear responses + source-memory task imagine-to-hear + digit-span mean span + (1 | site). The model *df* was 524 (Satterthwaite approximation).

## Discussion

In a general-population sample of 1,394 participants, we showed that hallucinatory experiences were associated with false perceptions and a lower response criterion on an auditory signal detection paradigm (Hypothesis 4) and with adverse childhood experiences (Hypothesis 5). However, hallucinatory experiences were not linked to impaired source memory, dichotic listening, or verbal working memory (Hypotheses 1–3). Additionally, we provided evidence that with these cognitive tasks, data quality from online recruitment is equal to that collected in the lab (Hypothesis 6). Our findings raise important issues regarding (a) continuities and discontinuities in hallucinatory experiences across the general population and in psychosis and (b) reproducibility in hallucinations research and in cognitive and clinical psychology.

### Continuity and discontinuity in hallucinatory experiences

Combined with previous evidence using auditory signal detection tasks (e.g., Barkus et al., 2011) and other paradigms aimed at assessing top-down influences on perception (de Boer et al., 2019; Vercammen & Aleman, 2010), this study provides strong evidence that hallucinatory experiences are associated with performance on the signal detection task, albeit with a small effect size. This finding held across both primary and secondary assessments of hallucinatory experiences and, in combination with results of previous studies, can be taken to support theoretical arguments regarding overweighted top-down processes in hallucinatory experiences (Powers et al., 2016). Increased false-alarm rates have been reported across a number of domains in schizophrenia (e.g., recognition memory; Weiss et al., 2004), although evidence comparing tasks across symptoms or task modality is lacking and should be a focus of further research. The evidence regarding sensitivity (ability to distinguish between speech and noise) was more equivocal; there was a very small association between hallucinatory experiences and *d*′, with CIs including 0—yet equivalence testing did not indicate that the effect was equivalent to 0. This highlights the extent to which precise parameter estimates require large samples; to our knowledge, this is the largest study to use the signal detection task alongside assessments of hallucinatory experiences, yet it is still not possible to confidently rule out a small impairment in sensitivity. We also found evidence for the contribution of adverse childhood experiences to hallucinatory experiences, consistent with previous evidence both in psychosis patients (Bailey et al., 2018) and in the general population (Lataster et al., 2006).

The findings were unequivocal, however, in showing no association between hallucinatory experiences and dichotic listening, source memory, and verbal working memory performance; effects were statistically indistinguishable from 0. This fails to conceptually replicate previous studies and suggests important complexities regarding the continuum hypothesis as applied to hallucinations. It also raises the question of how to interpret clinical findings in light of these results. In the case of dichotic listening, meta-analytic evidence supports the existence of a reduced right-ear advantage (Ocklenburg et al., 2013) and poorer performance on the forced-attention conditions (Hugdahl et al., 2013) in schizophrenia patients with hallucinations. A meta-analysis by Brookwell et al. (2013) reported that source-monitoring errors were specifically associated with hallucinations in psychosis and hallucinatory experiences in the general population. This study, in contrast, provides evidence that no such association exists in the general population. These results advance our understanding of the underlying cognitive mechanisms of hallucinatory experiences, importantly including those that seem not to be important in the general population. One potential interpretation is that there is a discontinuity in the mechanism between clinical and nonclinical hallucinations; that is, atypical language lateralization, poor attentional control, or source-monitoring biases may be markers of clinically significant hallucinations but not less frequent or less distressing experiences. That said, clinical studies on the cognitive mechanisms of hallucinations often use small sample sizes and nonstandardized methods, and direct replications are rare. It is therefore not clear how well these results would replicate if subjected to large-scale preregistered studies in patient populations. In addition, individuals with hallucinations of similar intensity to patients but without apparent distress or disability (e.g., the nonclinical hallucinators reported by Powers et al., 2017, and Sommer et al., 2010) have been an important comparison group. Further preregistered studies with large samples in these groups are needed to clarify whether these mechanisms are continuous across nonclinical and clinically significant hallucinations.

### Reproducibility in hallucinations research

In terms of reproducibility, these results may be a cause for concern in hallucinations research (and cognitive and clinical psychology more broadly). Of the five hypotheses regarding hallucinatory experiences, this study supported only two, despite previous evidence for all five. Poor reproducibility has been reported across psychology (Camerer et al., 2018), but as other researchers have noted, steps such as making data, code, and materials openly available and preregistering studies are likely to improve the field (Button et al., 2013). The reproducibility crisis has not been directly addressed in this area. In this study, we aimed to take a first step in addressing the issue.

A key part of the present study involved collecting data at sites across the world and online. We used three attention checks, excluding participants who failed more than one (following Peer et al., 2017). There was a negligible difference between the proportion of participants excluded because of attention-check failure in the lab-based and online data, providing evidence that online participants were equally as engaged as lab-based participants while reflecting a more diverse demographic. There were only negligible differences in effect sizes between lab-based and online data. This study, therefore, provides support for the feasibility of collecting cognitive task data online, which is of similar quality to data collected in the lab.

### Limitations and directions for future research

There are a number of limitations that should be considered when drawing conclusions from the present study. First, although the authors of this article collectively decided that the four cognitive tasks reported here were of the highest importance, they represent only a small selection of domains that may be important; other candidates for inclusion were intentional inhibition of memories (Waters et al., 2003) and meta-cognition (Varese & Bentall, 2011). Even the tasks we selected have multiple variants, for example, priming participants during the signal detection task to enhance the top-down component (Vercammen & Aleman, 2010) or increasing cognitive load in a source-memory task (Woodward et al., 2007). Task variation could be an important factor underlying inconsistency in the literature, as some tasks may be closer to relevant theoretical concepts than others (e.g., variation in self-generation of words in source memory). It is possible that task variations could account for the null effects reported here. Further research should investigate task manipulations affecting the association between performance and hallucinatory experiences. Second, the CAPS provided skewed data; comparatively few participants scored very high, potentially weakening the ability to detect associations with cognitive tasks. That said, our use of the LSHS-E as a secondary measure, which provides less skewed data and a higher prevalence of endorsed items, suggested an almost identical pattern of correlations. Third, scales such as the CAPS or LSHS-E do not provide separate assessments of different modalities of hallucinatory experience (e.g., auditory, visual, tactile). In future work, researchers should investigate these using specific assessments (or individual items) for different modalities. Finally, although we recruited participants native to 46 different countries, the data-collection sites themselves were situated mainly in western European countries. Future studies could, therefore, expand to include more culturally diverse countries and expand the multisite approach to further cognitive domains in clinical and nonclinical populations.

## Supplemental Material

sj-pdf-1-pss-10.1177_0956797620985832 – Supplemental material for Correlates of Hallucinatory Experiences in the General Population: An International Multisite Replication Study Click here for additional data file. Supplemental material, sj-pdf-1-pss-10.1177_0956797620985832 for Correlates of Hallucinatory Experiences in the General Population: An International Multisite Replication Study by Peter Moseley, André Aleman, Paul Allen, Vaughan Bell, Josef Bless, Catherine Bortolon, Matteo Cella, Jane Garrison, Kenneth Hugdahl, Eva Kozáková, Frank Larøi, Jamie Moffatt, Nicolas Say, David Smailes, Mimi Suzuki, Wei Lin Toh, Todd Woodward, Yuliya Zaytseva, Susan Rossell and Charles Fernyhough in Psychological Science

## Transparency

*Action Editor:* Daniela Schiller

*Editor:* Patricia J. Bauer

*Author Contributions*

S. Rossell and C. Fernyhough are joint last authors of this article and contributed equally to this study. P. Moseley, D. Smailes, S. Rossell, and C. Fernyhough developed the study concept. All the authors contributed to the study design and supervised a data-collection site or collected data. J. Moffatt programmed all task measures. P. Moseley analyzed and interpreted the data and drafted the manuscript. All the authors commented on, edited, and approved the final manuscript for submission.

**Open Practices:** The full data set, task code, analysis code, and self-report measures have been made publicly available via OSF and can be accessed at https://osf.io/eqy76. The design and analysis plans for the study were preregistered on AsPredicted; a copy of the preregistration is available at https://osf.io/cyu6j. Deviations from the preregistered analysis plan are discussed in the main text. This article has received the badges for Open Materials and Preregistration. More information about the Open Practices badges can be found at http://www.psychologicalscience.org/publications/badges.
