# Supplementary material for: Correlates of Hallucinatory Experiences in the General Population: An International Multisite Replication Study
Source: Psychol Sci. 2021 Jun 4;32(7):1024–37. doi: 10.1177/0956797620985832 (PMC8641136; doi:10.1177/0956797620985832)
Supplement: sj-pdf-1-pss-10.1177_0956797620985832 – Supplemental material for Correlates of Hallucinatory Experiences in the General Population: An International Multisite Replication Study [file sj-pdf-1-pss-10.1177_0956797620985832.pdf]

Correlates of hallucinatory experiences in the general population: an international multi-site replication study – supplemental materials

S1. Further details on the headphone check

For online data collection, participants were required to pass a short headphone check task before gaining access to the main task platform. The task is designed such that it can only be successfully completed if the participant is wearing headphones. The participant completes 6 trials, each comprising of 3 sequentially presented pure tones (200Hz, duration = 1000ms), with one tone presented at a lower volume than the others, and with one of the tones presented 180° out of phase. The task is designed so that when played through loudspeakers, the tones are indistinguishable, yet when played through headphones, the quietest tone is easily detected. The participant is required to select which of the tones is the quietest, and 'passes' the task if they correctly choose the quietest tone on at least 5/6 trials (which would occur at a rate of approximately 1.8% by chance). As part of the check, participants were also presented with two short bursts of noise to each ear, and asked to ensure that the headphones were positioned the correct way round (for the dichotic listening task). No data was gathered from participants who did not proceed past the headphone check.

S2. Further details on the attention checks

The first question, within the demographics section, stated: "*The following question is an attentional check. Please do not answer the sports question - just click the 'continue' button, below, to move on to the next part of the study. Which sport do you like?*". Participants were provided with a list of sports to choose from, but only passed the attention check if they did not answer. The second question, embedded within the CAPS questionnaire, stated "*While watching TV, have you ever had a fatal heart attack?*", with participants failing by selecting 'yes'. The third question, placed at the end of the study, stated "*The questions below are an attention check. Please select "Other" and type "Memory" into the text box. Once you have done that, click "continue" to complete the study! What was this study about?*", followed by a number of options, as well as the option to type into a free-text box. Participants failed this check if they did not correctly select "Other" and type into the text-box as instructed.

S3. Exclusions based on pre-registered criteria

CAPS - A further 4 participants were excluded based on a high number of missed responses to the CAPS scale (> 33%), or due to unfeasibly fast response to questions on the CAPS (< 1.5s per item).

Source memory - 13 participants were excluded from the source memory data, meeting one or more of the pre-registered exclusion criteria: due to 100% task accuracy (n = 1), scoring at or below 33.3% accuracy (n = 9), or scoring at or below 50% on old-new accuracy (n = 5).

Dichotic listening - 126 participants were excluded from the dichotic listening task performance, based on Bless et al. (2015), due to scoring < 50% accuracy on homonymous trials in any of the three task conditions (n = 83) and/or scoring a laterality index of 100% to one ear (n = 44).

Digit span - 32 participants were excluded from the backwards digit span data, scoring a mean span ≤ 3 (*n* = 26) or ≥ 12 (*n* = 6). One further participant was excluded because data from the digit span task did not save due to a technical error

Auditory signal detection - 11 participants were excluded from the auditory signal detection task data, scoring a d' of ≤ 0 (indicating at or below chance performance) (*n* = 10), and/or a hit rate of ≤ 10% (*n* = 3).

Adverse childhood experiences - data on adverse childhood experiences was excluded if participants completed ≤ 66% of the questionnaire (*n* = 19), or spent < 1.5s per item (*n* = 14). In total, 22 participants' data was excluded from the ACE.

S4. Source memory - further analysis

Exploratory (non-preregistered analysis) showed that there was a very small correlation between old-new accuracy and CAPS score (r_s = 0.09, 95% CI = [0.04, 0.14], *p* <0.001), suggesting that increased reports of hallucinations were weakly associated with a higher performance on recognition memory. However, this association was not found when the LSHS-E was used as a measure of hallucinations (r_s = 0.01, 95% CI = [-0.04, 0.06], *p* = 0.704). One further analysis strategy has been to adjust performance for guessing by subtracting the number of new words misrecalled as heard from the number of imagined words recalled as heard (Woodward & Menon, 2011). This adjusted variable did not correlate with CAPS score (r_s = .017, *p* = .537).

S5. Dichotic listening - task performance analysis

In the non-forced attention condition, as expected an overall right ear advantage was evident; that is, participants were more likely to correctly identify the syllable presented to their right ear (*M* = 13.06, 95% CI = [12.85, 13.26]) than the syllable presented to their left ear (*M* = 10.59, 95% CI = [10.41, 10.77]) (*t*(1267) = 14.56, *p* < .001, *d* = 0.41). In the forced right condition, participants were more likely to correctly report the syllable presented to their right ear (*M* = 16.86, 95% CI = [16.58, 17.14]) than the syllable presented to their left ear (*M* = 7.59, 95% CI = [7.38, 7.8]) (*t*(1267) = 39.49, p < .001, *d* = 1.11). In the forced left condition, participants were more likely to correctly report the syllable presented to the left ear (*M* = 15.52, 95% CI = [15.23, 15.80]) than the syllable presented to the right ear (*M* = 8.24, 95% CI = [8.01, 8.47]) (*t*(1267) = -29.52, *p* < .001, *d* = -0.83).

S6. Comparison of lab and online data

The below table provides means for important variables, split into lab and online data.

Variable	Lab data (M, SD)	Online data (M, SD)	Cohen's *d*	95% CI (mean diff)
Age	27.17 (11.0)	31.04 (10.56)	0.36	[-5.02, -2.72]
Gender (% female)	65.5	48.5		
Diagnosis (%)	15.7	16.3		
CAPS score	4.69 (4.79)	4.66 (4.81)	0.01	[-0.479, 0.542]
SMT – imagine-to-hear errors	4.04 (2.85)	4.04 (2.90)	0.00	[-0.308, 0.305]
DL – non-forced right-ear syllables	12.92 (3.72)	13.17 (3.78)	0.07	[-0.670, 0.163]
DS – mean span	6.42 (1.49)	6.37 (1.57)	0.03	[-0.117, 0.211]
ACE – total score	1.49 (2.24)	1.95 (2.54)	0.19	[-0.712, -0.204]

To test whether there was any divergence between findings from data collected in the lab and data collected online, we repeated the analyses for H1-5 for lab data only and online data only, with no difference in results: there was no association between CAPS and dichotic listening, digit span, or source memory performance, but there was an association between CAPS score and adverse childhood experiences, in both sets of data. There was also no difference between the number of exclusions due to failed attention checks in the lab data (3.86%) as compared to the online data (4.73%), (χ^2 = 0.48, p = .488).

S7. List of data collection sites, and number of participants from each site included in the full dataset, after exclusion criteria were applied.

Data collection sites: University of Bergen, Norway; University of Cambridge, UK; Durham University, UK; University of Groningen, Netherlands; Kings College London, UK; University of Montpellier, France; National Institute for Mental Health, Czech Republic; University of Roehampton, UK; Swinburne University, Melbourne, Australia; University College London, UK; University of British Columbia, Vancouver, Canada. Data collection sites have been assigned codes to preserve anonymity.

**Data collection site**	***N***
S1	41
S2	43
S3	78
S4	36
S5	52
S6	52
S7	54
S8	66
S9	78
S10	43
S11	51
Online data collection	800
**TOTAL**	**1394**

S8. Model comparison for linear mixed models reported in section 3.9.

AIC = Akaike Information Criteria. X2 and *p* values refer to the comparison to the previous model.

**Model name**	**Simpler model compared against**	**Fixed effects added**	**Random effect**	**AIC**	**X2**	***p***
**Random effects only**	-	None	Site	488.0	-	-
**Baseline**	Random effects only	Age, gender, parental income	Site	477.4	16.6	< .001
**Signal detection**	Baseline	Signal detection false alarms	Site	474.6	4.8	.028
**All variables**	Signal detection	Dichotic listening correct right ear responses, source memory imagine-hear errors, mean digit span	Site	480.3	0.27	.965

S10. Rates of self-reported psychiatric or neurological diagnoses in full sample (N = 1394). Note that there is overlap in diagnoses (i.e., some participants reported multiple diagnoses).

**Diagnosis**	***N***
Major depressive disorder	92
Generalised anxiety disorder	63
Post-traumatic stress disorder	14
Bipolar disorder	13
Attention-deficit hyperactivity disorder	9
Borderline personality disorder	7
Autism spectrum disorder	6
Schizophrenia/schizo-affective disorder/psychosis	5
Obsessive compulsive disorder	5
Eating disorder	5
Migraine	5
Dyslexia	4
Epilepsy	3
Learning disability	1
Body dysmorphia	1
Dyspraxia	1
Multiple sclerosis	1
Specific diagnosis not disclosed or unclear	41

S11. Histograms of task and self-report variables

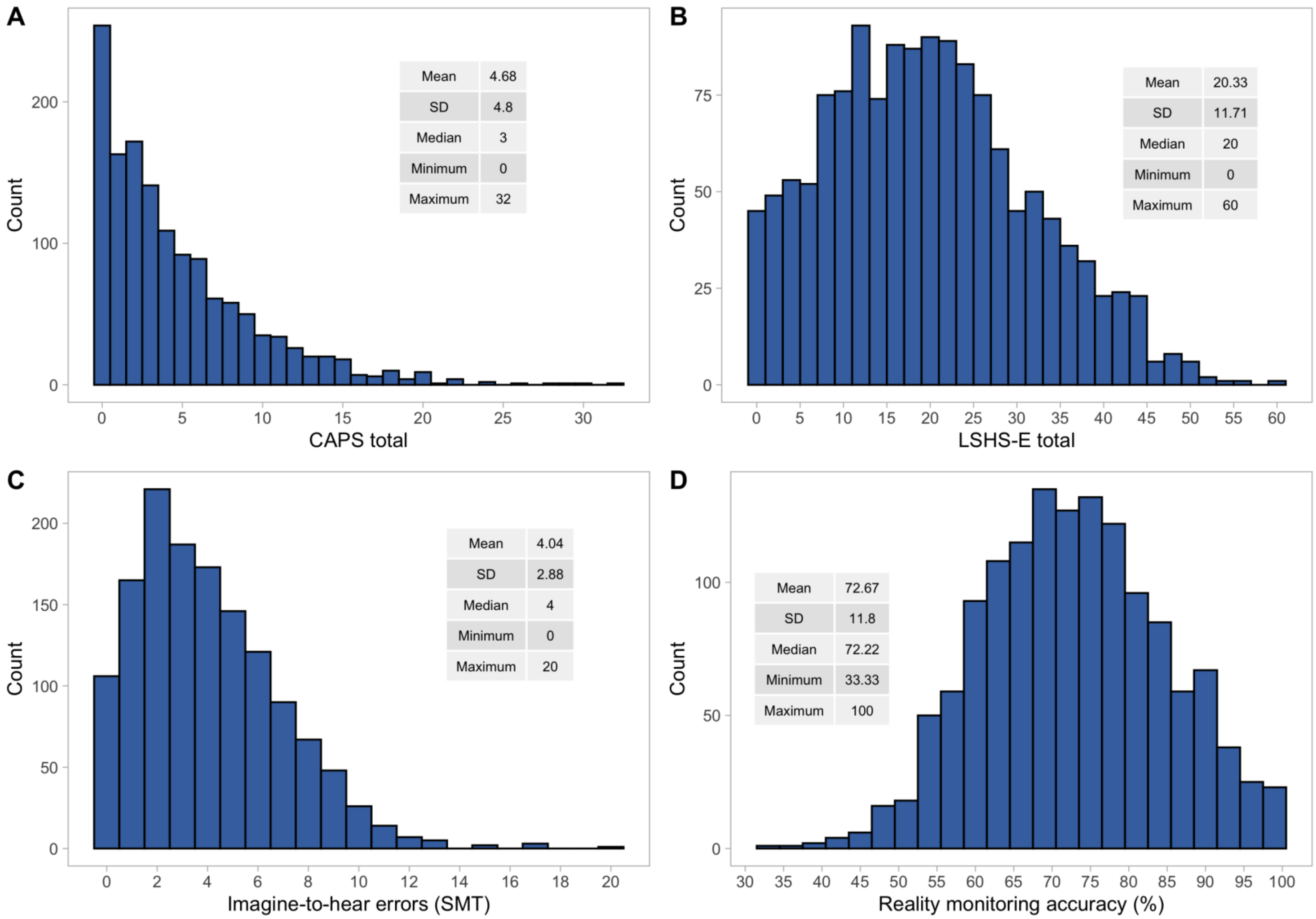

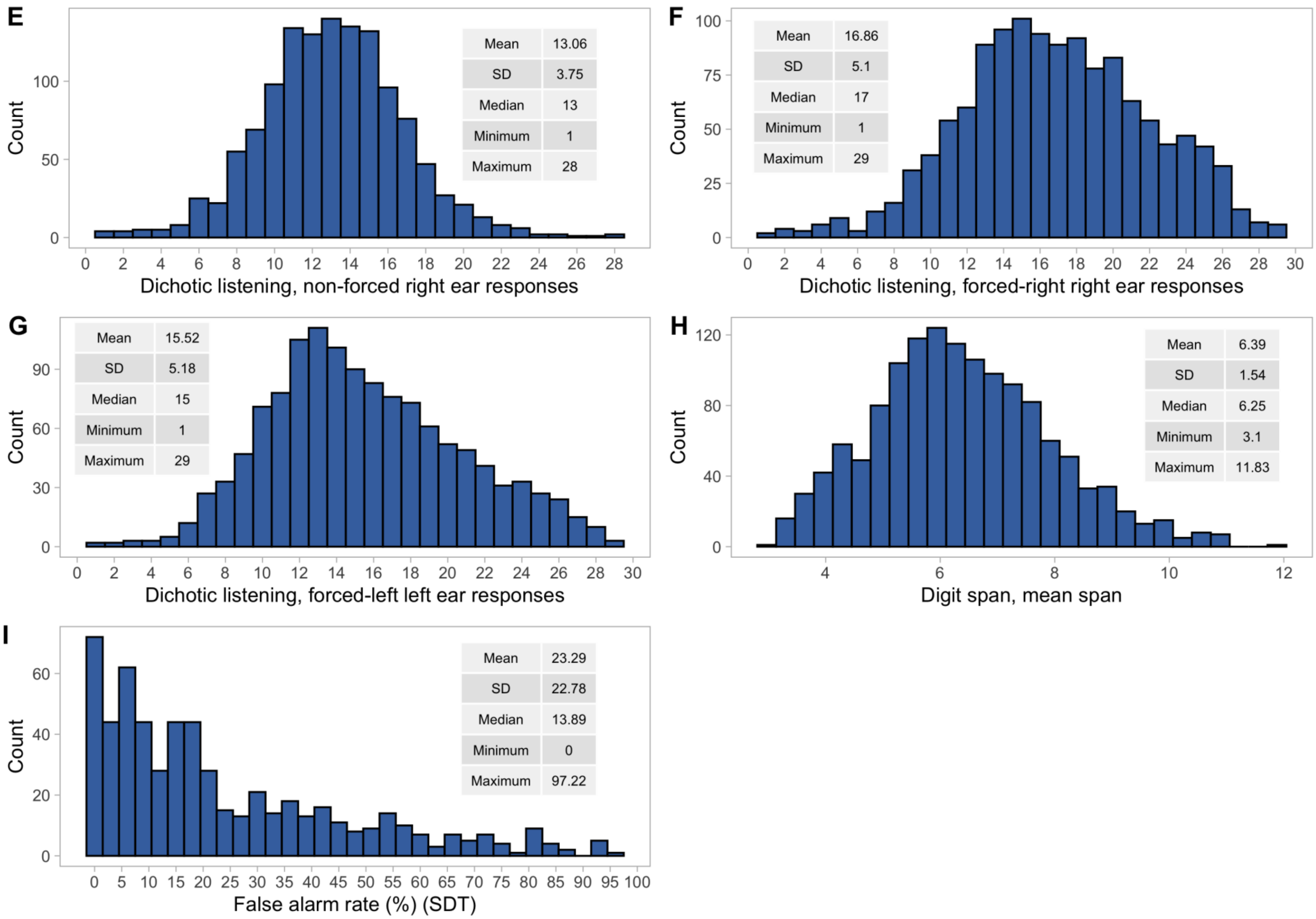

E
Count
Dichotic listening, non-forced right ear responses
Mean 13.06
SD 3.75
Median 13
Minimum 1
Maximum 28
F
Count
Dichotic listening, forced-right right ear responses
Mean 16.86
SD 5.1
Median 17
Minimum 1
Maximum 29
G
Count
Dichotic listening, forced-left left ear responses
Mean 15.52
SD 5.18
Median 15
Minimum 1
Maximum 29
H
Count
Digit span, mean span
Mean 6.39
SD 1.54
Median 6.25
Minimum 3.1
Maximum 11.83
I
Count
False alarm rate (%) (SDT)
Mean 23.29
SD 22.78
Median 13.89
Minimum 0
Maximum 97.22
